# Enhanced micropollutant degradation over catalyst-free synergistic activation of periodate and persulfate under solar light

**DOI:** 10.1007/s11356-025-36020-3

**Published:** 2025-02-06

**Authors:** Zexiao Zheng, Justin H. K. Man, Xiaoying Wang, Alvin S. K. Kwan, Kwan To Yim, Irene M. C. Lo

**Affiliations:** 1 Department of Civil and Environmental Engineering, The Hong Kong University of Science and Technology, Hong Kong, China; 2 Institute for Advanced Study, The Hong Kong University of Science and Technology, Hong Kong, China

**Keywords:** Micropollutant degradation, Periodate, Peroxydisulfate, Solar light driven, Synergistic activation

## Abstract

Micropollutants are ubiquitous in water sources, posing threats to both human health and ecosystems. Conventional water and wastewater treatment processes are inefficient in micropollutant removal. In this study, the energy-effective and environmentally friendly solar light–driven periodate (PI) and peroxydisulfate (PDS) synergistic activation process (PI/PDS/solar light) is developed for efficient micropollutant decontamination. The PI/PDS/solar light process (0.5 mM PI and 0.25 mM PDS) achieves 100% degradation of 2 ppm CBZ in 15 min with a CBZ degradation rate constant of 0.31 min^−1^, which is 6.6 and 13.2 times that of PI/solar light (0.046 min^−1^, 0.5 mM PI) and PDS/solar light (0.023 min^−1^, 0.5 mM PDS). Mechanistic studies reveal that the enhanced solar light utilization and charge transfer between PI and PDS lead to the synergistic activation of the dual oxidants in the PI/PDS/solar light process, thus promoting micropollutant degradation. Additionally, the scavenging tests demonstrate that ^**•**^OH and SO_4_^**•**−^ are the dominant radicals for CBZ degradation. Furthermore, the PI/PDS/solar light process exhibits excellent applicability in different types of water sources, where several water components (pH, natural organic matter, and anions) pose insignificant impacts on CBZ degradation. Nonetheless, the developed process still has a disadvantage in that the degradation intermediates of PPCPs may bring potential toxicity. The study offers valuable mechanistic insights into the novel synergistic PI and PDS coactivation process under solar light and highlights the practicability of the developed technique as an efficient strategy for micropollutant decontamination.

**Supplementary Information:**

The online version contains supplementary material available at 10.1007/s11356-025-36020-3.

## Introduction

Micropollutants are a diverse range of anthropogenic chemicals that occur in aquatic environments with concentrations at trace levels (ng/L to µg/L), which can cause detrimental impacts on human health and ecosystems (Barbosa et al. 2016). Micropollutants contain both natural compounds (e.g., antibiotics, estrogens) and synthetic chemicals (e.g., fluorinated surfactants), which are widespread in different water bodies and persistent in the environment (Yang et al. 2021a, b; Kumar et al. 2023). They are discharged into the environment through human activities, and the main sources are various industries, wastewater treatment plants (WWTPs), and agriculture (Luo et al. 2014). Currently, both conventional water and wastewater treatment processes are powerless to remove micropollutants (Ratchnashree et al. 2023). Therefore, it is paramount that novel techniques are developed to efficiently eradicate micropollutant contamination, thereby eliminating its adverse effects.

In recent years, advanced oxidation processes (AOPs) have been widely developed for micropollutant degradation (Kulišťáková 2023). Typically, Fenton and Fenton-like processes, employing liquid-form H_2_O_2_ as the oxidant to produce the reactive hydroxyl radical (^•^OH) for micropollutant oxidation, have attracted increasing attention (Thomas et al. 2021; Yan et al. 2021). Nevertheless, the limitations, such as the acidic operation conditions (pH 3–5), intensive production of ion sludge, and difficult transportation and stockpiling of H_2_O_2_ solution, hinder the applications of these techniques in wastewater treatment (Ziembowicz and Kida 2022). Alternatively, novel AOPs using solid-form oxidants, such as periodate (PI) and peroxydisulfate (PDS), provide efficient approaches for micropollutant removal with wide pH operational ranges and high safety (for chemical transportation and storage) (Duan et al. 2020; Du et al. 2020). Developing highly efficient activators for these solid-form oxidants in order to initiate micropollutant degradation is greatly needed.

Generally, ultrasound, heat, UV light, transition-metal-based nanoparticles, and liquid transition metal ions, have been developed as efficient activators of PI (Li et al. 2022a, b; Niu et al. 2022) and PDS (Hassani et al. 2023; Ding et al. 2021), but they face the challenges of being energy-intensive (ultrasound, heat, and UV light) or causing possible secondary pollution (metal leaching, toxic products from reacting with water matrices, etc.) (Rayaroth et al. 2022). Solar light, containing UV, visible, and infrared light, is safe, green, and inexhaustible, and solar energy has been widely utilized in different research fields, such as green H_2_ evolution, wastewater treatment, and CO_2_ to fuel conversion (Guo et al. 2021; Zheng et al. 2021a, b; Albero et al. 2020). Recently, solar light is also considered an ideal activator of PI and PDS (Yang et al. 2021a, b). Liu et al. recently reported that simulated solar light can efficiently activate PI (PI/solar light) to generate different reactive species, including hydroxyl radical (^•^OH, 1.8–2.7 eV vs. RHE), iodyl radical (^•^IO_3_), and superoxide radical anion (O_2_^•−^), for water disinfection (Liu et al. 2022). Another study found that PI can be activated by solar light to produce IO_3_^•^ as the dominant reactive species for the degradation of highly toxic PPD-quinone. However, the PI/solar light system is inefficient in micropollutant degradation because PI was found only to respond to the UV region of solar light (UV accounts for only 5% of the overall solar spectrum), leading to an insufficient radical yield (Zhang et al. 2021). Additionally, the produced reactive species in PI/solar light possess low redox potential for contaminant oxidation. On the other hand, Wen et al. found that PDS can be activated by the visible part (visible light accounts for 45% of the solar spectrum) of the solar light (PDS/visible light) to produce SO_4_^•−^ as the dominant reactive species for organic pollutant degradation (Wen et al. 2022). Nevertheless, the reported process is highly chemical-intensive (5.0 mM PDS) due to the low reactivity of PDS to visible light.

The coactivation of PI and PDS under solar light illumination (PI/PDS/solar light) may be a promising strategy to address the limitations of PI/solar light and PDS visible light in micropollutant degradation. First, the coactivation of PI and PDS can produce highly oxidative SO_4_^•−^ (2.7–3.1 eV vs. RHE) for efficient micropollutant oxidation, addressing the limitation of PI/solar light with low oxidation ability (Zheng et al. 2023a). Second, PI activation is an electron-donating process that can promote PDS activation to reduce chemical consumption because PDS can be activated by accepting the generated electrons during PI activation (Liu et al. 2022; Li et al. 2024). Third, the PI and PDS coactivation can enhance the utilization of the solar spectrum (PI for UV and PDS for visible light) for higher radical yields. Therefore, the PI/PDS/solar light process is expected to perform very efficiently in micropollutant degradation because of the improved radical yield and production of highly reactive SO_4_^•−^. To our best knowledge, the coactivation of PI and PDS by solar illumination has not been studied for micropollutant degradation, and thus the investigation of PI and PDS coactivation by solar illumination for efficient micropollutant degradation is of great importance.

Herein, a novel strategy of the synergistic activation of PI and PDS driven by solar light is developed to efficiently degrade micropollutants. To this end, the study focuses on the following aspects: (i) testing the efficiency and feasibility of the PI/PDS/solar light system in degrading micropollutants; (ii) revealing the mechanisms of the synergistic activation of PI and PDS by solar light for micropollutant degradation; and (iii) assessing the practicability of the PI/PDS/solar light system by operating the process in different types of water sources and investigating water matrix effects. The study offers an innovative approach to utilize solar energy for enhancing the coactivation of PI and PDS to enhance the reactive species yield for the efficient remediation of micropollutant contamination.

## Experimental

### Materials and chemicals

Potassium periodate (KIO_4_, 99.8%), potassium peroxydisulfate (K_2_S_2_O_8_, ≥ 99.0%), potassium peroxomonosulfate (KHSO_5_, ≥ 99.0%), hydrogen peroxide (H_2_O_2_, 35%), carbamazepine (CBZ, C_15_H_12_N_2_O, ≥ 99.0%), ibuprofen (IBU, C_13_H_18_O_2_, ≥ 98.0%), benzophenone (BZP, C_14_H_12_O_3_, ≥ 99.0%), bisphenol A (BPA, C_15_H_16_O_2_, ≥ 99.0%), sulfamethoxazole (SMX, C_10_H_11_N_3_O_3_S, ≥ 99.0%), sodium sulfate (NaSO_4_, ≥ 99.0%), sodium nitrate (NaNO_3_, ≥ 99.0%), sodium chloride (NaCl, ≥ 99.0%), *p*-benzoquinone (*p*-BQ, C_6_H_4_(= O)_2_, ≥ 98.0%), phenol (C_6_H_6_O, ≥ 99.0%), and isopropanol (IPA, C_3_H_8_O, ≥ 99.7%) were obtained from Sigma-Aldrich (St. Louis, MO, USA). Methanol (CH_3_OH, 99.9%) and acetonitrile (CH_3_CN, 99.9%) in high-performance liquid chromatography (HPLC)-grade and phosphate acid (H_3_PO_4_, ≥ 99.0%) were purchased from Merck (Darmstadt, Germany). All of the chemical reagents were used directly without further purification.

### Experimental setup and procedures

The performance of the developed PI/PDS/solar light system was studied using several micropollutants, including CBZ, IBU, SMX, BZP, and BPA, as the target contaminants. The chosen micropollutants are persistent in conventional wastewater treatment processes and have complex chemical structures. Therefore, the developed system would have wide applicability for degrading most micropollutants if it can efficiently degrade the above-mentioned contaminants. The experimental setup for contaminant degradation consists of a 300-W xenon lamp (Perfect Light Technology, Beijing) with a PLS-AM1.5G filter to provide simulated solar light for the reactions, a magnetic stirrer to ensure good mixing of the solution and chemical reagents, and a quartz reactor to support the reactions of contaminant degradation. All micropollutant degradation experiments were performed using this setting unless otherwise indicated.

The methodological flow diagram of the research work is shown in Figure S1, including the performance tests, mechanism investigation, and practicability evaluation. Typically, a 50-mL mixture containing 1 ppm target contaminants and 0.5 mM PI and 0.25 mM PDS was prepared for the PI/PDS/solar light process. The initial micropollutant concentration was set as 1 ppm to simulate the sum concentrations of various micropollutants in surface water. If the PI/PDS/solar light can efficiently remove such a concentration of micropollutants, it is practicable in the real world for water treatment. The contaminant degradation was initiated by irradiating the mixture under simulated solar light. As a comparison, the control experiments using PI/solar light, PDS/solar light, and PI/PDS were conducted without PDS addition, PI addition, and solar light illumination, respectively (Table S1). At predetermined time intervals, 1 mL of water samples were collected using a syringe and quenched with Na_2_S_2_O_3_ solution to terminate the contaminant degradation. The concentrations of the target PPCPs were analyzed using high-performance liquid chromatography (HPLC, Agilent, USA) equipped with a Waters symmetry C18 column and a UV detector. The mobile phase for detecting CBZ, BPA, IBU, and BZP consisted of water (using phosphoric acid to adjust to pH 2.0) and methanol (volume ratio 70%:30%) at a flow rate of 1.0 mL min^−1^, while the SMX measurement consisted of water (using phosphoric acid to adjust to pH 3.0) and acetonitrile (volume ratio 60%:40%) as the mobile phase.

Several experiments were conducted to reveal the mechanisms of the PI/PDS/solar light process in micropollutant degradation. To reveal the solar spectrum for oxidant activation, the performance of the PI/visible light, PDS/visible light, and PI/PDS/visible light processes was evaluated using a xenon lamp with a UV cut-off filter (λ ≥ 420 nm) as the light source. A photoluminescence spectrophotometer (Hitachi F-4500, Japan) was applied to obtain the 3D Excitation-Emission-Matrix (EEM) spectra for investigating the destruction of CBZ by the PI/PDS/solar light process. An electrochemical workstation (Corrtest CS350M, Wuhan, China) was applied to investigate the charge transfer between PI and PDS in the PI/PDS/solar light process using the I-t measurement and to study the redox reactions during the oxidant activation with and without solar illumination using cyclic voltammetry (CV) methods. The reactive species scavenging tests were conducted to evaluate the contribution of different radicals (^**•**^OH, SO_4_^**•**−^, O_2_^**•**−^, and ^**•**^IO_3_) to CBZ degradation. In detail, p-BQ was used to scavenge O_2_^**•**−^, IPA was applied as the quencher of ^**•**^OH, ethanol was employed to quench ^**•**^OH and SO_4_^**•**−^, and phenol was added to remove ^**•**^OH, SO_4_^**•**−^, and ^**•**^IO_3_. To reveal the pathways of CBZ degradation in the PI/PDS/solar light process, the water samples were measured using a liquid chromatography equipped with a mass spectrometer (LC–MS, Agilent, USA) to determine the degradation intermediates of CBZ.

To analyze the practical potential of the PI/PDS/solar light system, micropollutant degradation experiments were carried out using various types of water sources, including deionized (DI) water, river water, tap water, and rainwater. To evaluate the applicability of the PI/PDS/solar system at various pH values, the reaction solutions were adjusted using 0.1 M of HCl and NaOH solutions to obtain the desired pH values from 5.0 to 9.0. To evaluate the effects of various water matrices, 1 mM of Cl^−^, SO_4_^2−^, HCO_3_^−^, and NO_3_^−^ was added to the reaction solution to study the effects from anions, and 0.5, 1, 2, and 3 ppm of Suwannee River natural organic matter (NOM) were used to determine the influence from NOM. The above experiment was performed in triplicate and the standard deviation of the data obtained was less than 5%, ensuring high reliability of the study.

## Results and discussion

### Performance study of the PI/PDS/solar light process

**Fig. 1:**
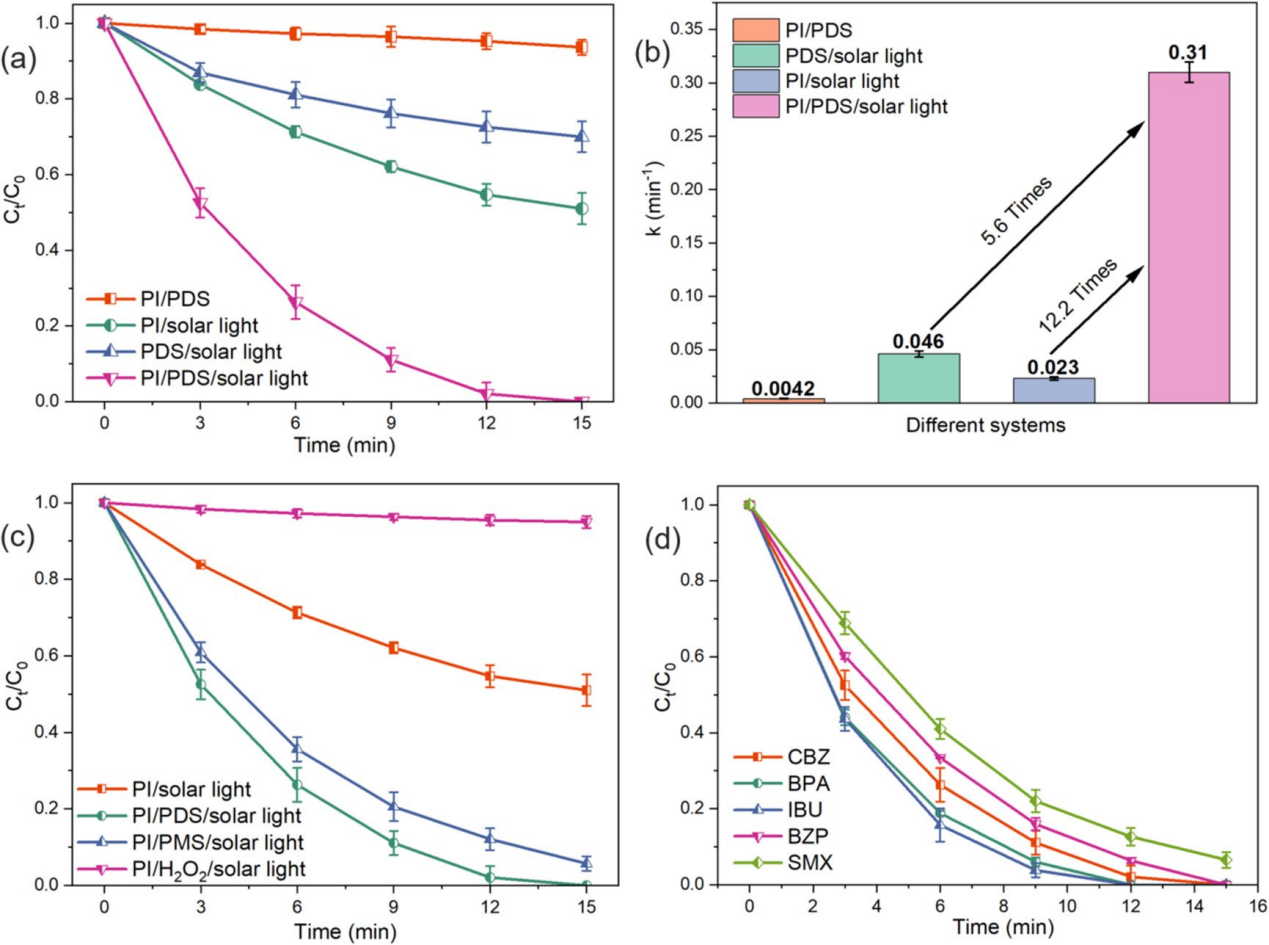
CBZ degradation **a** profiles and **b** rate constants by different processes; **c** CBZ degradation by coactivation of PI and different chemicals under solar light; **d** degradation of different micropollutants by the PI/PDS/solar light process. Experimental conditions: [CBZ]_0_ = [BPA]_0_ = [IBU]_0_ = [BZP]_0_ = [SMX]_0_ = 1 ppm; [PI]_0_ = 0.5 mM; [PDS]_0_ = [PMS]_0_ = [H_2_O_2_]_0_ = 0.25 mM for dual oxidants system; [PDS]_0_ = 0.5 mM for the PDS/solar light process; pH = 7.0 ± 0.1

The merits of PI and PDS coactivation were determined by investigating the CBZ degradation using various systems. As shown in Fig. 1a, without solar light, the PI/PDS shows negligible CBZ degradation, suggesting the activation of PI and PDS is initiated by solar light illumination. In the presence of sunlight, PI activation and PDS activation degrades 49.1% and 30.1% of CBZ in 15 min, respectively. The inefficient performance of these systems is mainly attributed to the insufficient yield of reactive species using a single oxidant. In comparison, the PI/PDS/solar light system achieves the best performance with 100% degradation of CBZ within 15 min. The CBZ degradation by PI/PDS/solar light process is compared to other processes with UV or solar light illumination (Table S2). Compared with other processes, the PI/PDS/solar light system exhibits advantages in achieving 100% CBZ removal with a short treatment duration and saving energy consumption by using renewable solar energy. The pseudo-first-order degradation rate constants of CBZ in different systems were determined to visualize the advantage of the PI/PDS/solar light process (Fig. 1b). The PI/solar light and PDS/solar light obtain CBZ degradation rate constants of 0.046 min^−1^ and 0.023 min^−1^, respectively. Most significantly, the PI/PDS/solar light system exhibits the highest rate constants of 0.30 min^−1^, achieving 5.6 times and 12.2 times increases compared with PI/solar light and PDS/solar light processes. The excellent performance in CBZ degradation by PI/PDS/solar light clearly demonstrates that the coactivation of PI and PMS achieves synergistic effects on CBZ degradation.

The coactivation of PI with various oxidants was compared to reveal the beauty of the configuration of PI/PDS. As shown in Fig. 1c, the PI/H_2_O_2_/solar light process shows insignificant CBZ degradation because H_2_O_2_ reacts with PI to form IO_3_^−^ to interfere with the oxidant activation process (Wen et al. 2022). The PI/PDS/solar light and PI/PMS/solar processes performed efficiently in CBZ degradation, obtaining 100% and 93.5% of CBZ degradation, respectively. Both PDS and PMS can be activated by accepting electrons from PI activation, leading to high CBZ removal efficiencies (Zheng et al. 2023a, b; Li et al. 2024). The coactivation of PI/PDS shows superior performance than PI/PMS since more SO_4_^•−^ can be generated from PDS activation (PDS: S_2_O_8_^2−^; PMS: HSO_5_^2−^), and thus PDS is a more suitable oxidant to be coactivated with PI. The feasibility of the PI/PDS/solar light system in degrading varying micropollutants was investigated using CBZ, BPA, IBU, BZP, and SMX as the target contaminants (Fig. 1d). One hundred percent of CBZ, BPA, IBU, and BZP can be removed by the PI/PDS/solar light process, while 93.4% of SMX is degraded, suggesting that the system has a high degradation efficiency for a wide range of micropollutants.

**Fig. 2:**
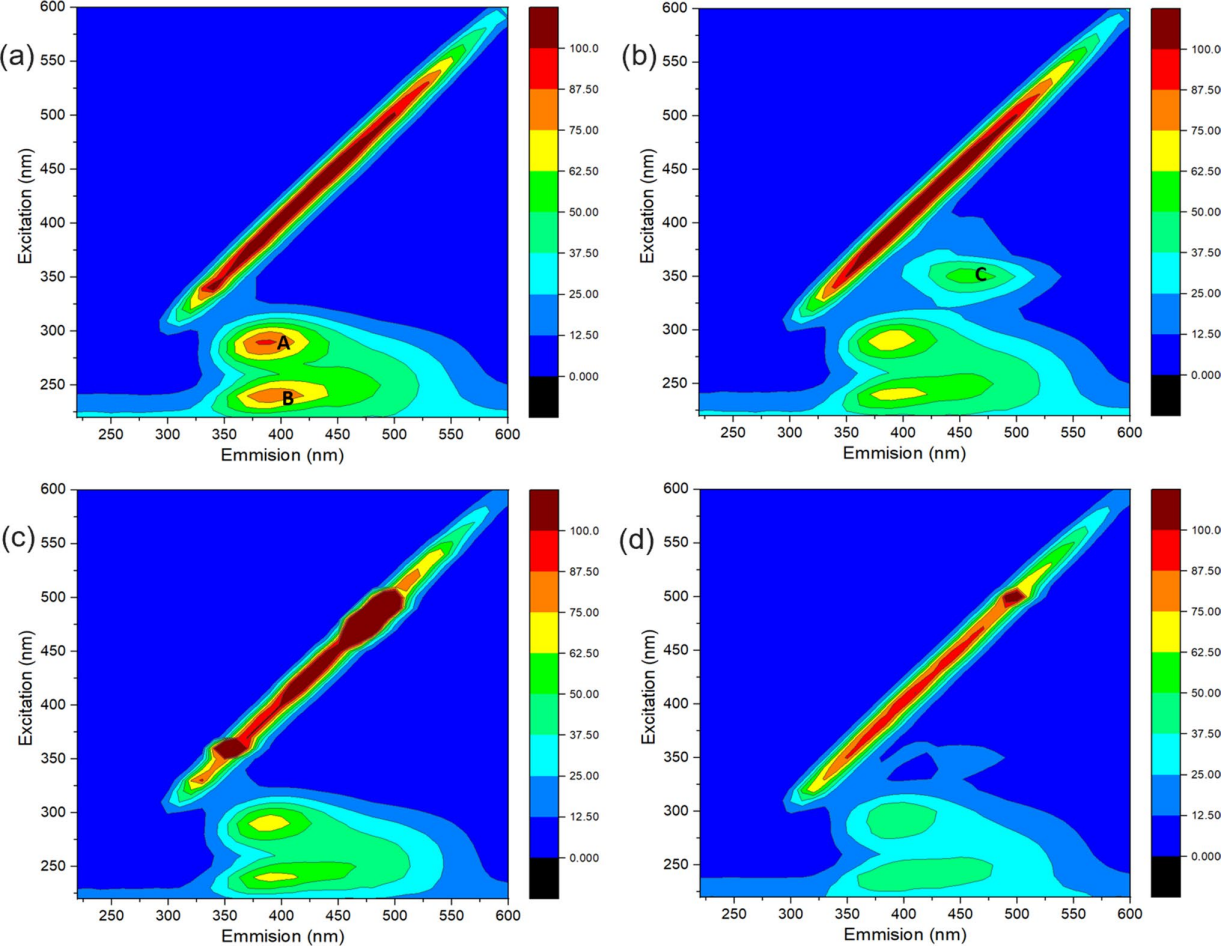
EEM measurements of CBZ solution **a** without treatment, and treated for 15 min by the **b** PDS/solar light process, **c** PI/solar light process, and **d** PI/PDS/solar light process

3D fluorescence Excitation-Emission-Matrix (EEM) spectra were employed to monitor the degradation behavior of CBZ over different systems. Figure 2a shows the EEM image of DI water spiked with 1 ppm CBZ before treatment. Two signals located at the fluorescence regions of excitation (Ex) 275–300 nm and emission (Em) 360–425 nm (letter A) and Ex 225–250 nm and Em 360–440 nm (letter B) are the characteristic peaks of CBZ (Pan et al. 2024). These peaks can be detected by fluorescence and are mainly attributed to the biphenyl ring structure on the CBZ molecular emitting absorption peaks with a strong intensity. As shown in Fig. 2b, after 15 min of treatment by the PDS/solar process, the intensities of CBZ characteristic peaks show a certain decrease, suggesting that a portion of CBZ is oxidized and results in the opening of the biphenyl ring structure. Meanwhile, an additional peak located at Ex 330–370 nm and Em 430–510 nm (letter C) can be observed after the treatment by the PDS/solar light process and is ascribed to the production of the fluorescent-respond intermediates during the CBZ oxidation by SO_4_^•−^. Similarly, the PI/solar light treatment also leads to a significant decrease in the intensities of the CBZ characteristic peaks (Fig. 2c). Different from the PDS/solar process, the PI/solar treatment does not form additive fluorescent-respond intermediates. Notably, as shown in Fig. 2d, the characteristic peaks of CBZ almost disappear after treatment by the PI/PDS/solar light system for 15 min, demonstrating that most of the CBZ molecules are oxidized and result in a particular amount of mineralization (Zheng et al. 2021a, b). The TOC removal was measured to determine the mineralization performance of CBZ degradation by different processes. As shown in Figure S2, the PI/PDS, PI/solar light, and PDS/solar light show low TOC removal efficiencies with 1.3%, 21.5%, and 12.4%, respectively. In comparison, the PI/PDS/solar light process achieves the best performance in mineralizing CBZ (52.3% TOC removal) among different processes, which is consistent with the highly efficient CBZ degradation achieved by this process. The above results confirm that the PI/PDS/solar light process demonstrates excellent performance in destroying the molecular structure of CBZ, thus achieving a better CBZ degradation performance and higher mineralization efficiency.

### Optimization of chemical dosages

**Fig. 3:**
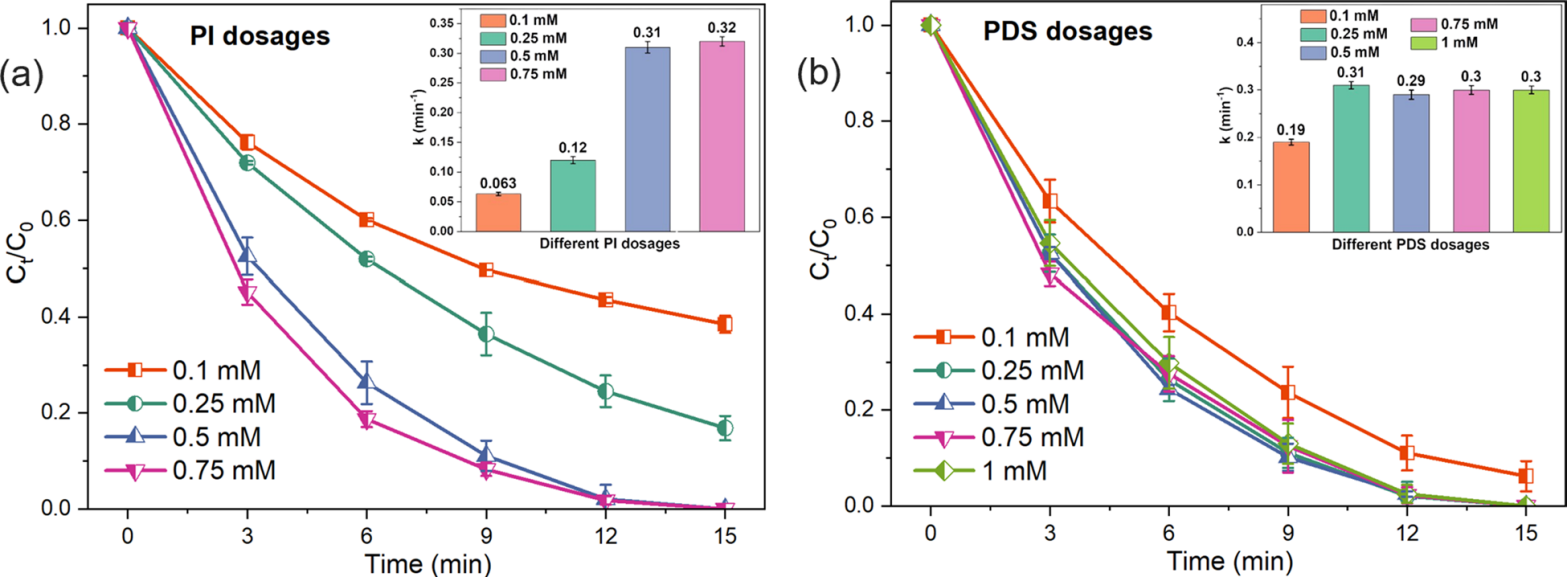
Effects of **a** PI dosages and **b** PDS dosages on CBZ degradation by the PI/PDS/solar light process. Experimental conditions: [CBZ]_0_ = 1 ppm; [PDS]_0_ = 0.25 mM for studying the effects of PI dosages; [PI]_0_ = 0.5 mM for studying the effects of PDS dosages; pH = 7.0 ± 0.1

The effects of PI and PDS dosages on CBZ degradation by the PI/PDS/solar light system were investigated to balance the CBZ degradation performance and chemical use. As shown in Fig. 3a, at a fixed PDS concentration of 0.25 mM, CBZ degradation efficiencies increase with the increase of PI dosages from 0.1 to 0.5 mM. One hundred percent removal of CBZ was obtained at both 0.5 mM and 0.75 mM within 15 min. The inset of Fig. 3a shows that the degradation rate constants of CBZ increase from 0.063 min^−1^ at 0.1 mM to 0.32 min^−1^ at 0.75 mM as more reactive species are generated due to the following: (i) at higher PI dosages, more IO_4_^−^ ions are available for activation to produce ^•^OH and ^•^IO_3_; and (ii) increased production of electrons during PI activation at higher PI dosages also promotes PDS activation to generate SO_4_^•–^ (Liu et al. 2022). In regard to the performance and cost-effectiveness, 0.5 mM PI was chosen for further investigation.

The effects of PDS dosages on CBZ degradation are different from that of PI dosages. As shown in Fig. 3b, at a fixed PI concentration of 0.5 mM, the PI/PDS/solar light removes 92.7% of CBZ at a PDS dosage of 0.1 mM, while complete degradation of CBZ is achieved at PDS dosages equal to or higher than 0.25 mM. Notably, the CBZ degradation rate constants increase from 0.19 min^−1^ at 0.1 mM PI to 0.31 min^−1^ at 0.25 mM PI, but they stabilize at around 0.30 min^−1^ when the PI dosage is equal to or higher than 0.25 mM. This stabilization of CBZ degradation rate constants indicates a threshold effect, which is mainly attributed to two reasons: (i) at PDS dosage higher than 0.25 mM, the electrons produced from PI activation are insufficient to activate the excess PDS (Li et al. 2022a); and (ii) the excess PDS in the aquatic solution can compete with CBZ for the available reactive species to interfere with the degradation process. To avoid the potential chemical waste, 0.25 mM was chosen as the optimal PDS dosage for the operation of the PI/PDS/solar light process.

### Mechanisms of synergistic activation of PI and PDS

#### Solar light utilization

**Fig. 4:**
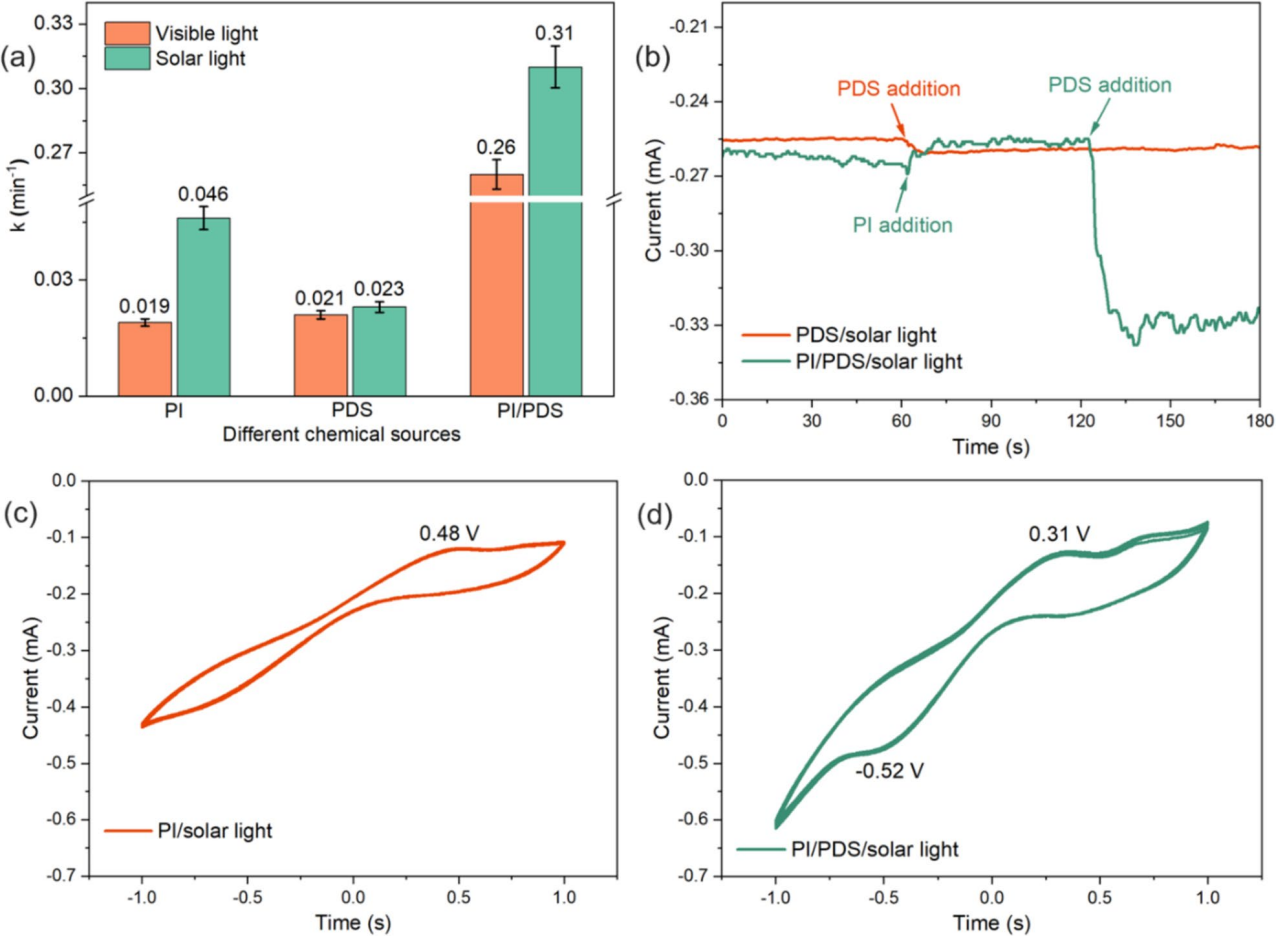
**a** Effects of light sources on CBZ degradation rate constants by the PI/solar light, PDS/solar light, and PI/PDS/solar light processes; experimental conditions: [CBZ]_0_ = 1 ppm; [PDS]_0_ = 0.25 mM; [PI]_0_ = 0.5 mM; pH = 7.0 ± 0.1; **b** I-t curves of the PDS/solar light, and PI/PDS/solar light processes; CV curves of **c** the PI/solar light and **d** PI/PDS/solar light processes

The synergistic activation of PI and PDS oxidants may be attributed to the improved utilization of the solar spectrum in the presence of dual oxidants. To confirm that the configuration of PI/PDS can facilitate the utilization of solar light, CBZ degradation by the activation of PI, PDS, and PI/PDS was conducted under visible light (≥ 420 nm, using a UV filter to block the UV part of the simulated solar light) as the light source. Figure 4a shows the CBZ degradation rate constants by different processes under visible light and solar light. For PI activation, the use of visible light results in a 58.7% decrease in the CBZ degradation rate constant compared with that of using solar light, indicating that the PI activation is mainly induced by the UV spectrum of the solar light. As a comparison, the obtained CBZ degradation rate constant by PDS/visible light (0.21 min^−1^) is almost the same as PDS/solar light (0.23 min^−1^). This result demonstrates that PDS activation is mainly triggered by the visible spectrum of the solar light due to the visible light having sufficient energy to decompose the O–O bond in the PDS molecules (Wen et al. 2022). As for the dual oxidant processes, the PI/PDS/visible light process achieves a CBZ degradation rate constant of 0.26 min^−1^ with only a 16.1% decrease compared to the PI/PDS/solar light process (0.31 min^−1^). Compared with PI activation, PI/PDS activation shows a lower performance drop in CBZ degradation when altering the light source, demonstrating that the introduction of PDS in the PI/PDS/solar light process significantly improves the utilization of solar light.

#### Charge transfer between PI and PDS

The charge transfer between the PI and PDS under solar light irradiation may also contribute to the synergistic activation of the dual oxidants. As shown in Figure S3, the scavenging of electrons using NaBrO_3_ from the PI/PDS/solar light process reduces the degradation efficiency of CBZ (from 100 to 68.4%) to a certain extent. This is mainly attributed to the elimination of electrons, which restrains PDS activation for reactive species formation. The I-t curves obtained from electrochemical characterization at an applied voltage of 0 V vs. Ag/AgCl under solar light illumination are used to analyze the charge transfer during PDS/PI activation. As shown in Fig. 4b, in the PDS/solar light process, the addition of PDS into the CBZ solution slightly decreases the system current (0.005 mA) due to the PDS activation by solar light being an electron-accepting process that captures electrons from the pollutants (Ren et al. 2020). As for the PI/PDS/solar light process, the addition of PI results in a slight current increase of 0.01 mA since PI activation under solar light illumination is an electron-donating process, generating a specific amount of electrons. Subsequently, the further addition of PDS into the system causes a notable current decrease of 0.077 mA, which is attributed to the charge transfer from PI to PDS. Notably, the current decrease by PDS addition in the PI/PDS/solar light process (0.077 mA) is much more significant than that in the PDS/solar light process (0.005 mA), demonstrating that the presence of PI in PI/PDS/solar light system can work as an electron donor to facilitate the activation of PDS. The above results suggest that the charge transfer between PI and PDS under solar light illumination is a crucial pathway that benefits the synergistic activation of PI and PDS.

Cyclic voltammetry (CV) measurements of the PI/solar light and PI/PDS/solar light processes were obtained to further investigate the redox reactions that occur during the activation of oxidants. The CV measurements were conducted under solar light illumination using the electrolytes in the presence of PI or PI/PDS, and the electrolytes being stirred magnetically. As shown in Fig. 4c, oxidation and reduction peaks of the PI/solar light process are observed at 0.48 V and − 0.64 V, respectively. In comparison, as shown in Fig. 4d, the PI/PDS/solar light process shows a lower oxidation peak (0.31 V) and reduction peak (− 0.48 V) than that of the PI/solar light process, indicating that redox reactions more readily occur under solar light in the presence of dual oxidants. Additionally, the PI/PDS/solar light process achieves a significantly higher current than the PI/solar light process, and is attributed to the coactivation of PDS and PI by solar light promoting charge transfer (Li et al. 2020). Overall, the above results reveal that the synergistic coactivation of PI and PDS under solar illumination can be mainly ascribed to the extended utilization of the solar spectrum and the improved charge transfer between PI and PDS.

#### Contributions of reactive species toward CBZ degradation

**Fig. 5:**
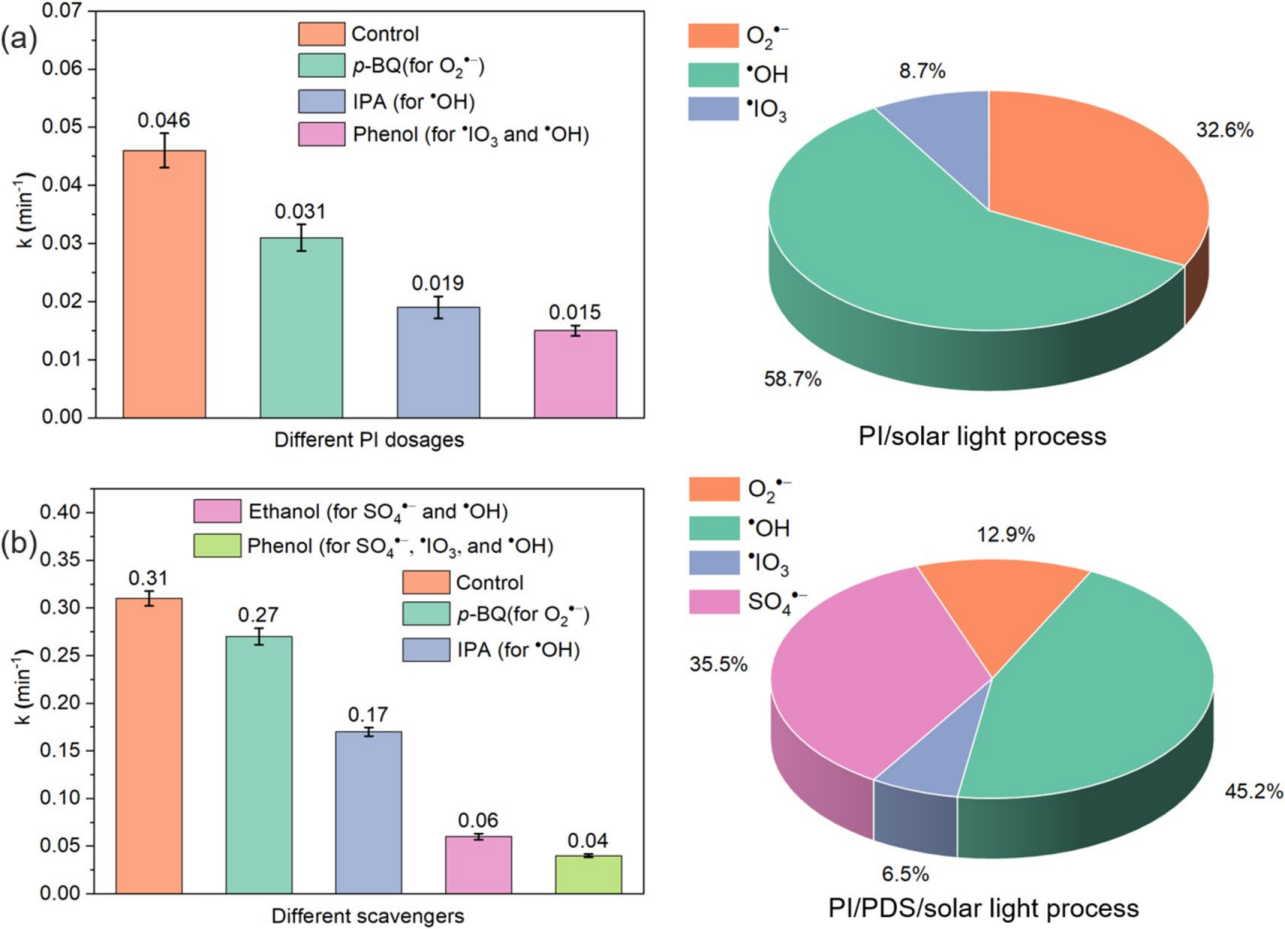
CBZ degradation rate constants in the conditions of adding different scavengers and contributions of reactive species to CBZ degradation by the **a** PI/solar light process and **b** PI/PDS/solar light process. Experimental conditions: [CBZ]_0_ = 1 ppm; [scavengers]_0_ = 0.1 mM; [PDS]_0_ = 0.25 mM; [PI]_0_ = 0.5 mM; pH = 7.0 ± 0.1

In the PI/solar light system, the PI (IO_4_^−^) can be activated by solar light to produce ^**•**^IO_3_ and O^**•**−^ radicals (Eq. 1), and O^**•**−^ can further react with H^**+**^ to form ^**•**^OH (Eq. 2) (Lee and Yoon 2004). Further, sufficient electrons are produced along with ^**•**^IO_4_ (weak reactive species that can not degrade micropollutants) in another PI activation pathway (Eq. 3). The produced electrons can reduce dissolved oxygen to further generate O_2_^**•**−^ (Eq. 4). Therefore, these reactive species, including ^**•**^OH, O_2_^**•**−^, and ^**•**^IO_3_, are likely to contribute to micropollutant degradation. The contributions of these reactive species to CBZ degradation in the PI/solar light process were evaluated by conducting scavenging tests using *p*-BQ, IPA, and phenol as the scavengers of O_2_^**•**−^, ^**•**^OH, and ^**•**^OH and ^**•**^IO_3_, respectively (Zheng et al. 2021a; Zhang et al. 2021). As shown in Fig. 5a, the addition of *p*-BQ and IPA suppresses the CBZ degradation rate constant by 0.015 min^−1^ and 0.027 min^−1^. Accordingly, the contributions of O_2_^**•**−^ and ^**•**^OH to CBZ degradation are determined as 32.6% and 58.7%, respectively, indicating that both O_2_^**•**−^ and ^**•**^OH are the dominant reactive species for CBZ degradation in the PI/solar light process. Meanwhile, the elimination of ^**•**^OH and ^**•**^IO_3_ by phenol reduces the CBZ degradation rate constant by 0.031 min^−1^. Thus, ^**•**^IO_3_ plays an insignificant role in CBZ degradation with a contribution of only 8.7%. Hence, the contributions of various radicals toward CBZ degradation in the PI/solar light process follow the sequence of ^**•**^OH > O_2_^**•**−^ > ^**•**^IO_3_.

1 $${{\text{IO}}_{4}}^{-}+hv\to {}^{\cdot }{\text{IO}}_{3}+{\text{O}}^{\cdot -}$$

2 $${\text{O}}^{\cdot -}+{\text{H}}^{+}\to {}^{\cdot }\text{OH}$$

3 $${{\text{IO}}_{4}}^{-}+hv\to {}^{\cdot }{\text{IO}}_{4}+{\text{e}}^{-}$$

4 $${\text{e}}^{-}+{\text{O}}_{2}\to {\text{O}}_{2}{}^{\cdot -}$$

As for the PI/PDS/solar light process, apart from the above-mentioned reactive species, SO_4_^**•**−^ may also be involved in CBZ degradation due to the presence of PDS (Eq. 5) (Maifadi et al. 2022). Figure 5b shows that the CBZ degradation rate constants are decreased to 0.27 min^−1^, 0.17 min^−1^, 0.07 min^−1^, and 0.04 min^−1^ with the addition of p-BQ, IPA, ethanol (scavenging ^**•**^OH and SO_4_^**•**−^), and phenol (scavenging ^**•**^OH, ^**•**^IO_3_, and SO_4_^**•**−^), respectively. Accordingly, the contributions of the involved radicals are determined as 45.2%, 35.5%, 12.9%, and 6.5% for ^**•**^OH, SO_4_^**•**−^, O_2_^**•**−^, and ^**•**^IO_3_, respectively. Compared with the PI/solar light process, the contributions from ^**•**^OH, O_2_^**•**−^, and ^**•**^IO_3_ show a certain decrease due to the generation of highly reactive SO_4_^**•**−^ accounting for a portion of CBZ degradation. Notably, the contribution of O_2_^**•**−^ significantly decreases from 32.6% in the PI/solar light process to 12.9% in the PI/PDS/solar light process. This is attributed to the electrons generated during PI activation in the PI/solar light process being mainly used for O_2_^**•**−^ production, while they are driven for PDS activation in the PI/PDS/solar light process, reducing the generated amount of O_2_^**•**−^ (Eq. 6) (Khan et al. 2024). On the other hand, the ^**•**^OH still maintains a high contribution to CBZ degradation in the PI/PDS/solar process because the activation of PDS can promote the generation of ^**•**^OH (Eq. 7) (Dong et al. 2021a, b). Overall, the scavenging tests confirm that the PI/PDS/solar light process produces the highly reactive SO_4_^**•**−^ for CBZ degradation, and thus significantly improves the performance.

5 $${\text{S}}_{2}{\text{O}}_{8}+hv\to {2\text{SO}}_{4}{}^{\cdot -}$$

6 $${\text{S}}_{2}{\text{O}}_{8}+{2\text{e}}^{-}\to {2\text{SO}}_{4}{}^{\cdot -}$$

7 $${\text{SO}}_{4}{}^{\cdot -} \, +{\text{H}}_{2}\text{O}\to {\text{ SO}}_{4}{}^{2-} \, +{}^{\cdot }\text{OH}+{\text{H}}^{+}$$

#### Degradation routes of CBZ

The degradation pathways of CBZ by the PI/PDS/solar light process were investigated using LC–MS measurement. As shown in Figure S4, 11 degradation intermediates of CBZ are identified. Accordingly, three degradation routes of CBZ are determined (Figure S5). In route I, the C-N bond of CBZ is oxidized by the ^•^OH to form the intermediate m/z = 210, which is then degraded by other radicals to generate m/z = 194. Route II begins with the oxidation of the C = C by the reactive oxygen species, producing m/z = 253a. Afterwards, m/z = 253a is further degraded through the oxidation of ^•^OH to yield m/z = 271 or other reactive oxygen species to cause the formation of m/z = 253b and m/z = 251. In route III, the reactive species induce the opening of the C═C bond to form dual C═O, forming m/z = 269. The further degradation of m/z = 269 involves intramolecular cyclization and aromatic substitution with m/z = 267 as a product. Then, the reactive species proceed with the contraction processes of m/z = 267, leading to the generation of m/z = 224, m/z = 196, and m/z = 180. Finally, these intermediates can be mineralized by the reactive species with CO_2_ and H_2_O as the main products. Overall, the CBZ degradation in the PI/PDS/solar light process mainly involves C–N bond cleavage, C═C bond deconstruction, hydroxylation, and contraction.

#### Overall mechanisms of the PI/PDS/solar light process

**Fig. 6:**
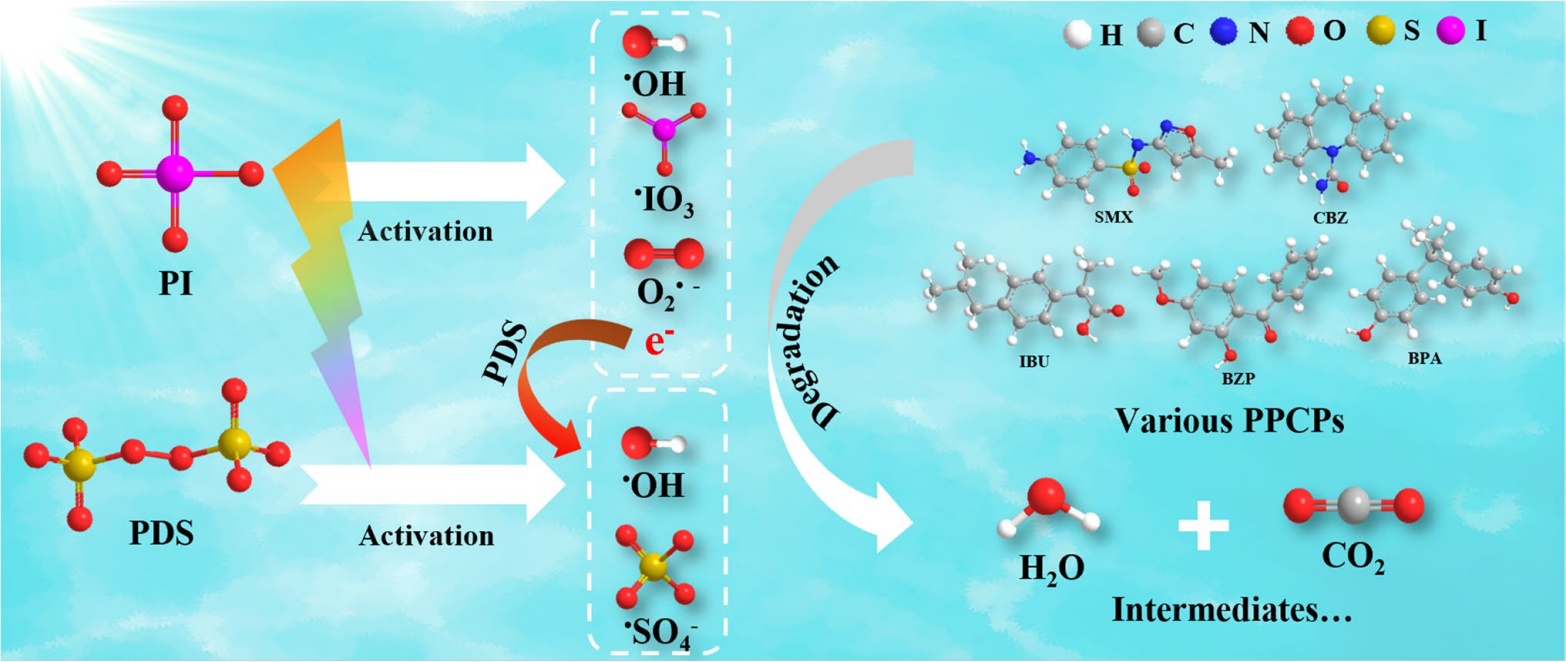
Schematic illustration of the micropollutant degradation by the PI/PDS/solar light process

The CBZ degradation mechanisms in the PI/PDS/solar light process are summarized in Fig. 6. Under the illumination of simulated solar light, PI can be activated by the UV spectrum to produce several radicals, including ^**•**^OH, ^**•**^IO_3_, and O_2_^**•**−^. PI activation also results in the generation of a certain number of electrons. Meanwhile, PDS can be activated by both the UV and visible light region of the simulated solar light and accepts the electrons produced from PI activation, leading to the formation of ^**•**^OH and SO_4_^**•**−^ radicals. All the generated radicals, including ^**•**^OH, SO_4_^**•**−^, O_2_^**•**−^, and ^**•**^IO_3_, can oxidize various micropollutants to be intermediates and ultimately mineralize them to CO_2_ and H_2_O, while ^**•**^OH and SO_4_^**•**−^ dominate the pollutant degradation. The reactive species cause the hydroxylation, deconstruction, and contraction of CBZ molecule, forming intermediates and eventually mineralizing CBZ. Overall, the coactivation of PI and PDS achieves a synergistic effect because the two oxidants make full use of the solar spectrum, and the PI activation process also produces electrons to promote PDS activation, leading to significantly increased yields of reactive species for micropollutant degradation.

### Practicability of the PI/PDS/solar chlorine process

**Fig. 7:**
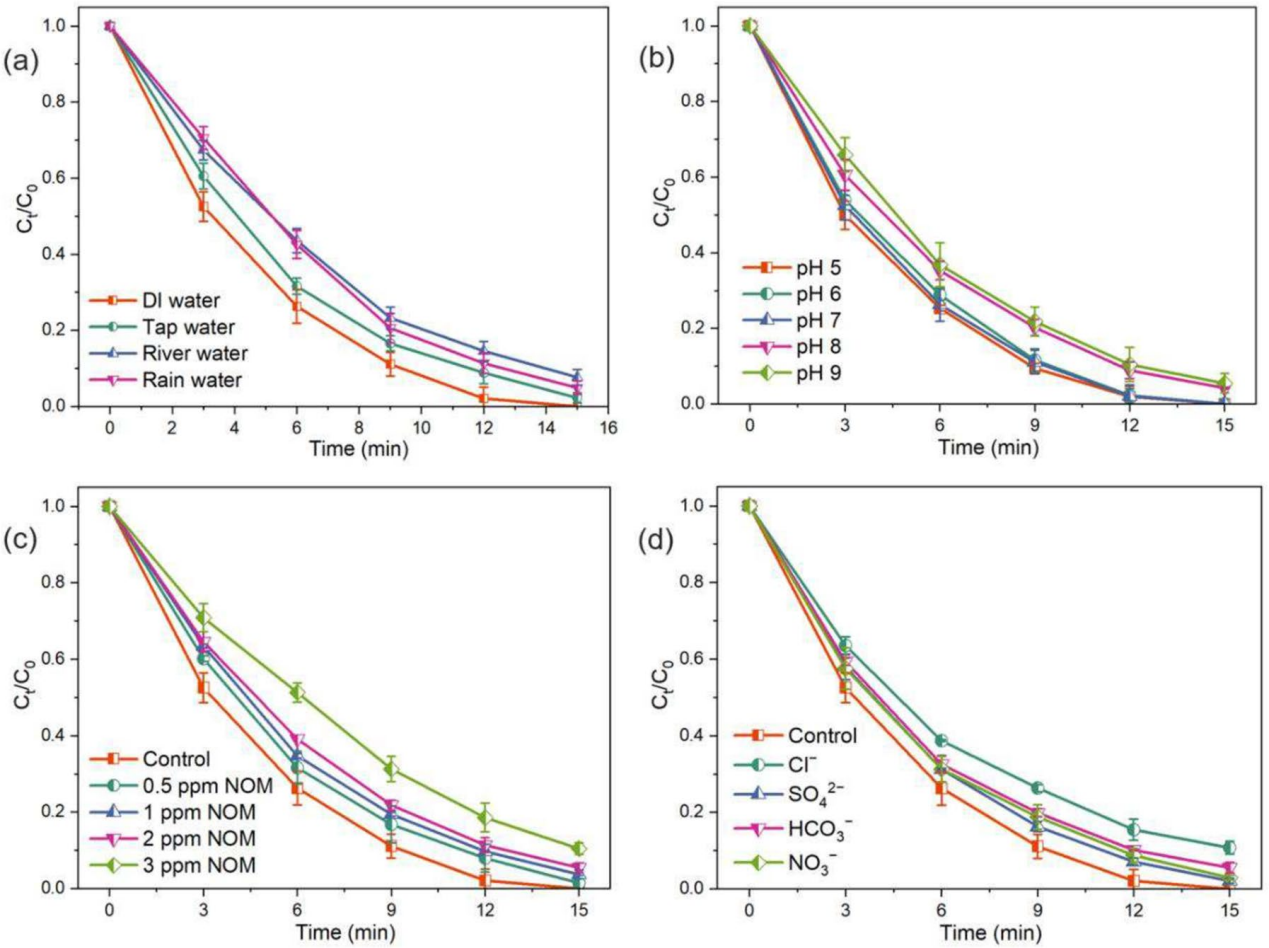
**a** CBZ degradation by the PI/PDS/solar light process in different types of water; effects of **b** pH, **c** NOM with different concentrations, and **d** different anions on CBZ degradation by the PI/PDS/solar light process. Experimental conditions: [CBZ]_0_ = 1 ppm; [PDS]_0_ = 0.25 mM; [PI]_0_ = 0.5 mM; [anions]Eq_0_ = 1 mM; pH = 7.0 ± 0.1 unless otherwise indicated

The practicability of the PI/PDS/solar chlorine process was evaluated by investigating its feasibility in treating different types of water sources and understanding the effects of various water matrices (e.g., pH, natural organic matters, and anions) on the process. The CBZ degradation performance of the PI/PDS/solar light process was tested using various types of real water samples, including DI water, tap water, river water, and rain water. As shown in Fig. 7a, the CBZ degradation efficiencies are 100%, 97.7%, 92.3%, and 95.1% for the tests using DI water, tap water, river water, and rain water. Compared with the test in DI water, the PI/PDS/solar light process shows decreased performance in the three real water samples. However, regardless of the type of water sample, the process can maintain at least 92% removal of CBZ, demonstrating that the PI/PDS/solar light system has a wide applicability for different real water samples. The reduced CBZ degradation performance in the real water samples may be ascribed to some water components interfering with PI/PDS activation for CBZ removal. Thus, we further studied the effects of various water matrices, mainly pH, natural organic matter, and anions.

#### Effects of pH

The effects of pH on the PI/PDS/solar light system were evaluated by testing CBZ degradation in the pH range of 5–9. As shown in Fig. 7b, the PI/PDS/solar light process achieves 100% removal of CBZ from pH 5 to 7 within 15 min and the CBZ degradation efficiencies decrease to 95.8% and 94.5% at pH 8 and 9. Accordingly, the CBZ degradation rate constants are around 0.31 min^−1^ at pH 5–7 and 0.21 min^−1^ at pH 8 and 9. Such results suggest that the PI/PDS/solar light exhibits outstanding performance in weak acidic and neutral conditions but is less efficient in alkaline conditions, which is consistent with a previous study of the UV/PDS process for pollutant removal (Jiang et al. 2023). Compared to the operation of the PI/PDS/solar light process in weak acidic and neutral conditions, two factors in alkaline conditions lead to reduced degradation efficiency of CBZ. First, most of the produced reactive species, including ^•^OH, SO_4_^•−^, and O_2_^•−^, have lower oxidation potentials for micropollutant degradation at alkaline conditions (Kumar et al. 2020). Second, the extensive existing OH^−^ in alkaline solution can quench the highly reactive SO_4_^•−^ (E° = 2.5–3.1 V) to generate ^•^OH (E° = 1.9–2.7 V) with low oxidation ability, thus delaying the degradation reactions (Dong et al. 2021a). Although decreased degradation efficiencies are recorded at higher pH, complete CBZ degradation can also be achieved by slightly increasing the duration of the PI/PDS/solar light process. Since most water and wastewater sources are within the pH range of 5–8, the good performance of the PI/PDS/solar light process guarantees its high practical potential.

#### Effects of NOM

Water and wastewater contain NOM, which is frequently reported to interfere with AOPs for organic pollutant removal. The effects of NOM on the PI/PDS/solar light process were investigated by spiking SRNOM with different concentrations in the solution. As shown in Fig. 7c, the CBZ degradation efficiencies by the PI/PDS/solar light process decrease with the increase of NOM concentrations from 0.5 to 3 ppm; particularly, 90.1% of CBZ is degraded at a NOM concentration of 3 ppm. It has been reported that the presence of NOM in suspensions can adsorb specific regions of the solar spectrum to reduce the intensity of solar light that can be used for PI and PDS coactivation, leading to lower generation of reactive species (Liu et al. 2022). Additionally, NOM can also consume a portion of the reactive species, thus reducing the availability of reactive species for CBZ degradation (Yang et al. 2021a). Therefore, decreased CBZ degradation performance is observed at high NOM concentrations. Nevertheless, the PI/PDS/solar light process can remove more than 90% of CBZ in the presence of 3 ppm NOM, whereas most water and wastewater sources possess lower NOM concentrations than this scenario, suggesting the system performs efficiently in the existence of NOM.

#### Effects of coexisting anions

The effects of various widely existing anions, including Cl^−^, SO_4_^2−^, HCO_3_^−^, and NO_3_^−^, with a concentration of 1 mM, on CBZ degradation were also studied. As shown in Fig. 7d, SO_4_^2−^, NO_3_^−^, and HCO_3_^−^ show insignificant influence on the CBZ degradation, where 98.3%, 97.1%, and 95.3% of CBZ can be removed, respectively. The negative effects of these coexisting anions on CBZ degradation are mainly caused by their quenching of the reactive species. SO_4_^2−^ can only react with SO_4_^•−^ and lead to a minor effect on CBZ degradation. Both NO_3_^−^ and HCO_3_^−^ can scavenge ^•^OH and SO_4_^•−^ to produce NO_3_^•^ and CO_3_^•−^ as products to restrain CBZ degradation. However, the reaction rates between these anions and reactive species are low, and thus slightly affect the CBZ degradation (Wang et al. 2022). In comparison, the presence of Cl^−^ greatly decreases CBZ removal efficiencies to 89.3% and 94.1%, respectively. Cl^−^ can efficiently scavenge a wide range of reactive species (e.g., ^•^OH, O_2_^•−^, and SO_4_^•−^) and produce reactive chlorine species with weak oxidation potentials toward organic pollutant degradation, leading to adverse effects (Zheng et al. 2023a, b). The above results suggest that the interference from SO_4_^2−^, NO_3_^−^, and HCO_3_^−^ is negligible, but Cl^−^ adversely affects CBZ degradation.

## Conclusions

This study reports an energy-effective and environmentally friendly PI/PDS/solar light process for efficient micropollutant degradation. The synergistic activation of PI and PDS under solar light illumination significantly improved the efficiency of micropollutant degradation, as evidenced by achieving 5.6 and 12.2 times increases in CBZ degradation rate constant compared to that of the PI/solar light and PDS/solar light processes. The improved utilization of solar light and the charge transfer between the dual oxidants lead to synergistic activation, thus promoting micropollutant degradation. Mechanism investigation reveals that ^**•**^OH and SO_4_^**•**−^ contribute the most to CBZ degradation in the PI/PDS/solar light process. Moreover, the PI/PDS/solar light process has good applicability, evidenced by its wide applicability in removing different micropollutants, good performance in various types of water sources, and insignificant interference from several water components. The study provides in-depth insights into the enhanced coactivation of PI and PDS under solar light illumination for micropollutant degradation, demonstrating an easily implementable, reality-relevant, and feasible technique for water treatment. Regarding the great potential of the process, further research may investigate the scaling up of the process for treating water with a large treatment volume in the real world and conduct the toxicity evaluation for the degradation intermediates of micropollutants and chemical usage.

## Supplementary Information

Below is the link to the electronic supplementary material.

Supplementary file1 (DOCX 763 KB)

## Data Availability

The authors confirm that the data supporting the findings of this study are available within the article.
