# Supplementary material for: Enhanced micropollutant degradation over catalyst-free synergistic activation of periodate and persulfate under solar light
Source: Environ Sci Pollut Res Int. 2025 Feb 6;32(28):16931–44. doi: 10.1007/s11356-025-36020-3 (PMC12325505; doi:10.1007/s11356-025-36020-3)
Supplement: Supplementary file 1 — Supplementary file1 (DOCX 763 KB) [file 11356_2025_36020_MOESM1_ESM.docx]

*Supplementary information for*

**Enhanced Micropollutant Degradation over Catalyst-Free Synergistic Activation of Periodate and Persulfate under Solar Light**

Zexiao Zheng^a^, Justin H.K. Man^a^, Xiaoying Wang^a^, Alvin S.K. Kwan^a^, Kwan To Yim^a^, Irene M.C. Lo^a, b*^

^a^ Department of Civil and Environmental Engineering, The Hong Kong University of Science and Technology, Hong Kong, China

^b^ Institute for Advanced Study, The Hong Kong University of Science and Technology, Hong Kong, China

* Corresponding author. Email address: cemclo@ust.hk; (Chair Professor Irene M. C. Lo); Tel.: +852 2358 7157; Fax: +852 2358 1534.

**The Supplementary materials include 10 Pages, 5 Figures, and 2 Tables.**


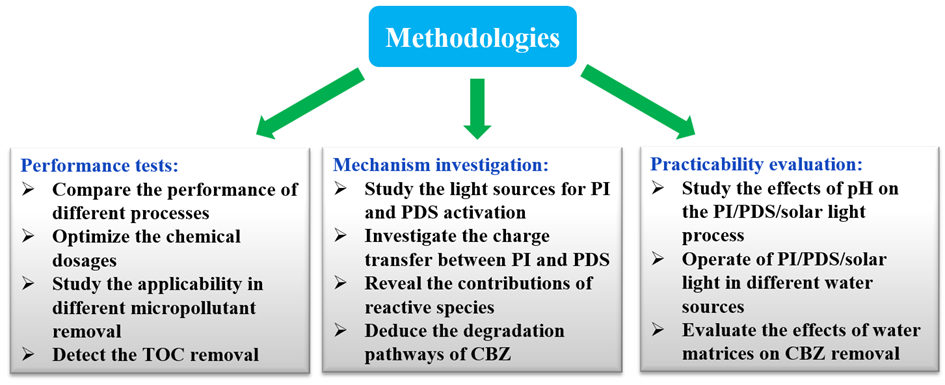
Figure S1. The methodological flow diagram of the research work


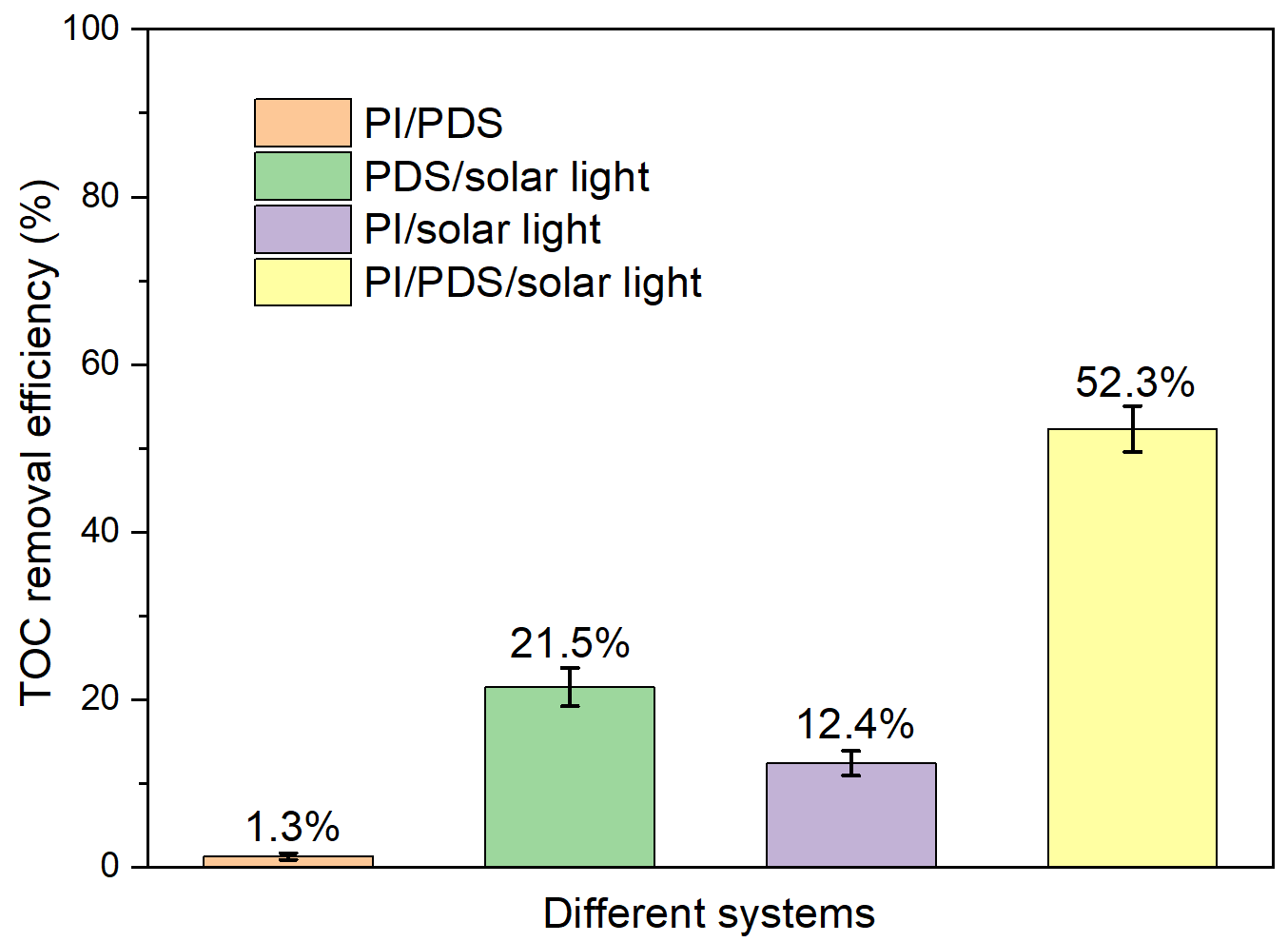
Figure S2. TOC removal efficiencies during the CBZ degradation by different processes. Experimental conditions: [CBZ]_0_ = 1 ppm; [NaBrO_3_]_0_ = 0.1 mM; [PDS]_0_ = 0.25 mM; [PI]_0_ = 0.5 mM; pH = 7.0 ± 0.1


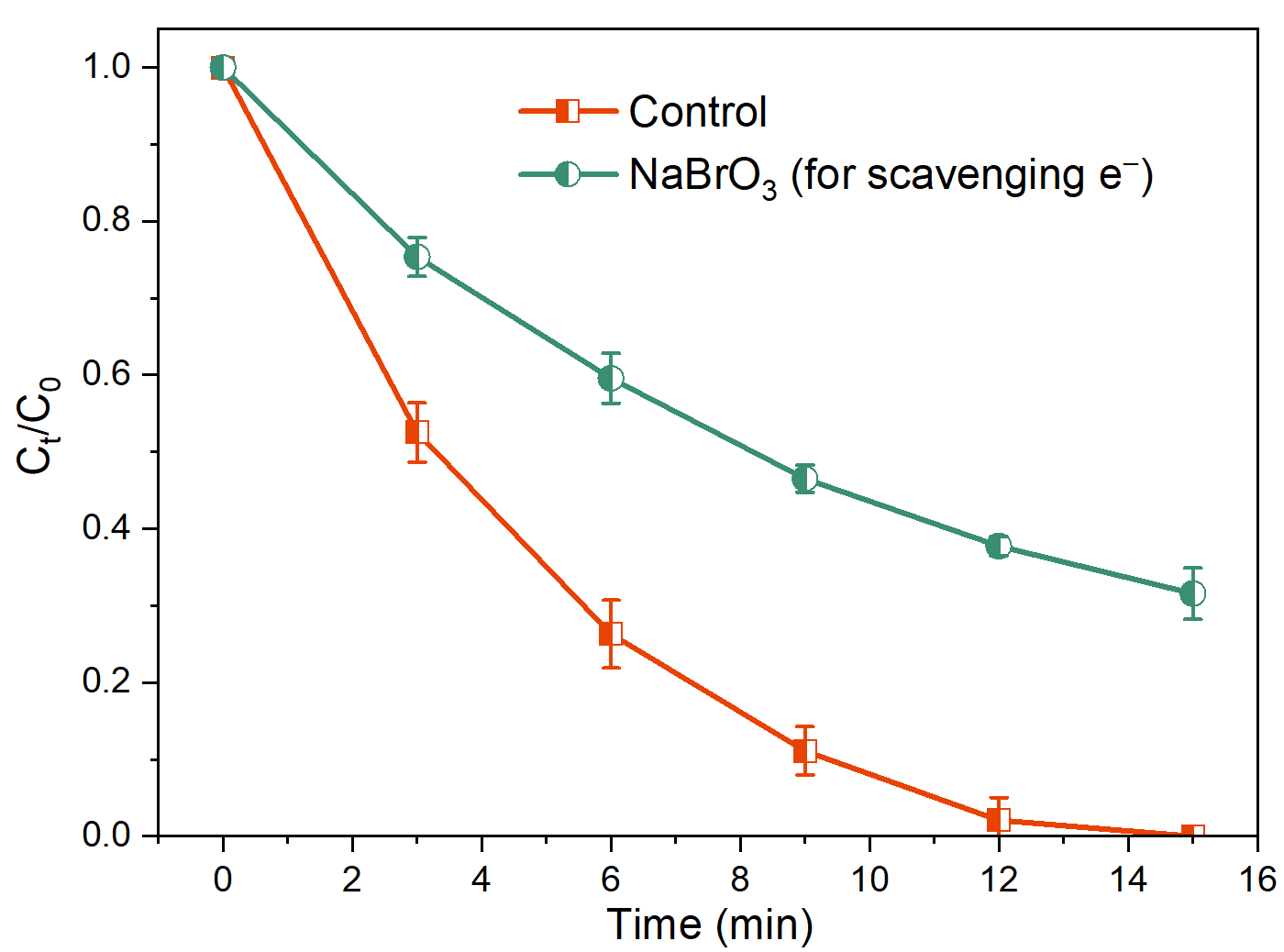
Figure S3. Effect of scavenging electrons on CBZ degradation by the PI/PDS/solar light process. Experimental conditions: [CBZ]_0_ = 1 ppm; [NaBrO_3_]_0_ = 0.1 mM; [PDS]_0_ = 0.25 mM; [PI]_0_ = 0.5 mM; pH = 7.0 ± 0.1


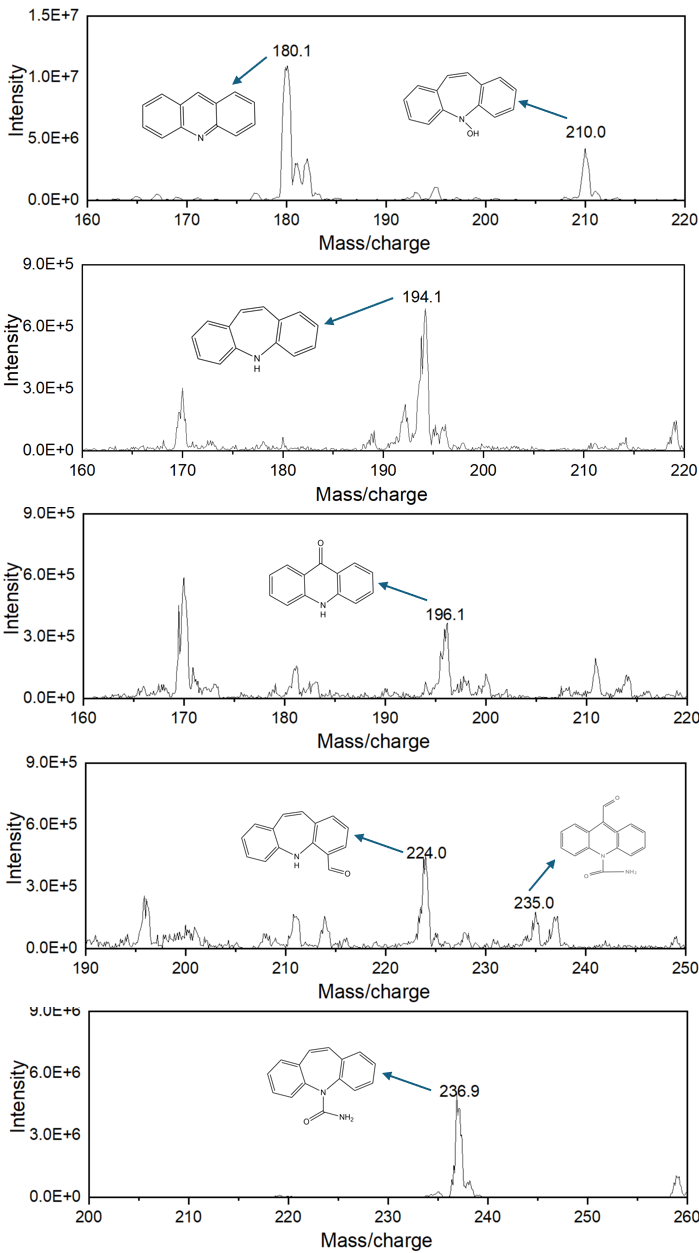


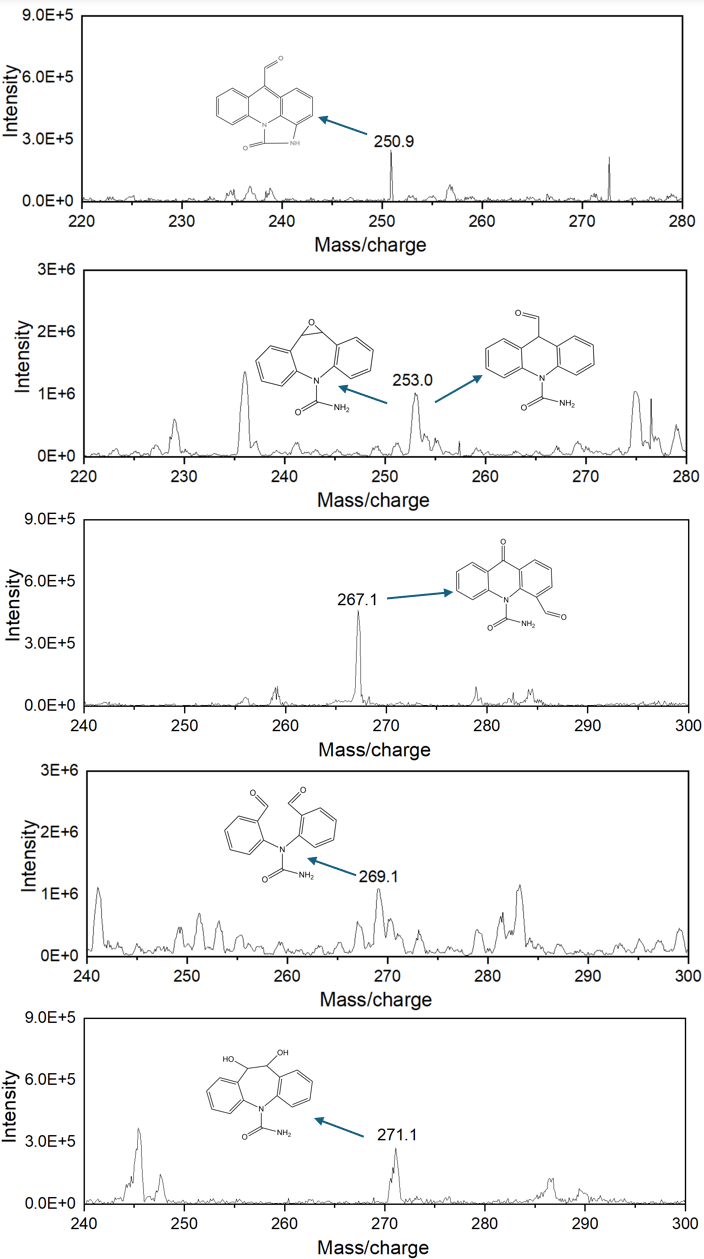
Figure S4. LC-MS patterns of intermediates during CBZ degradation by the PI/PDS/solar light process


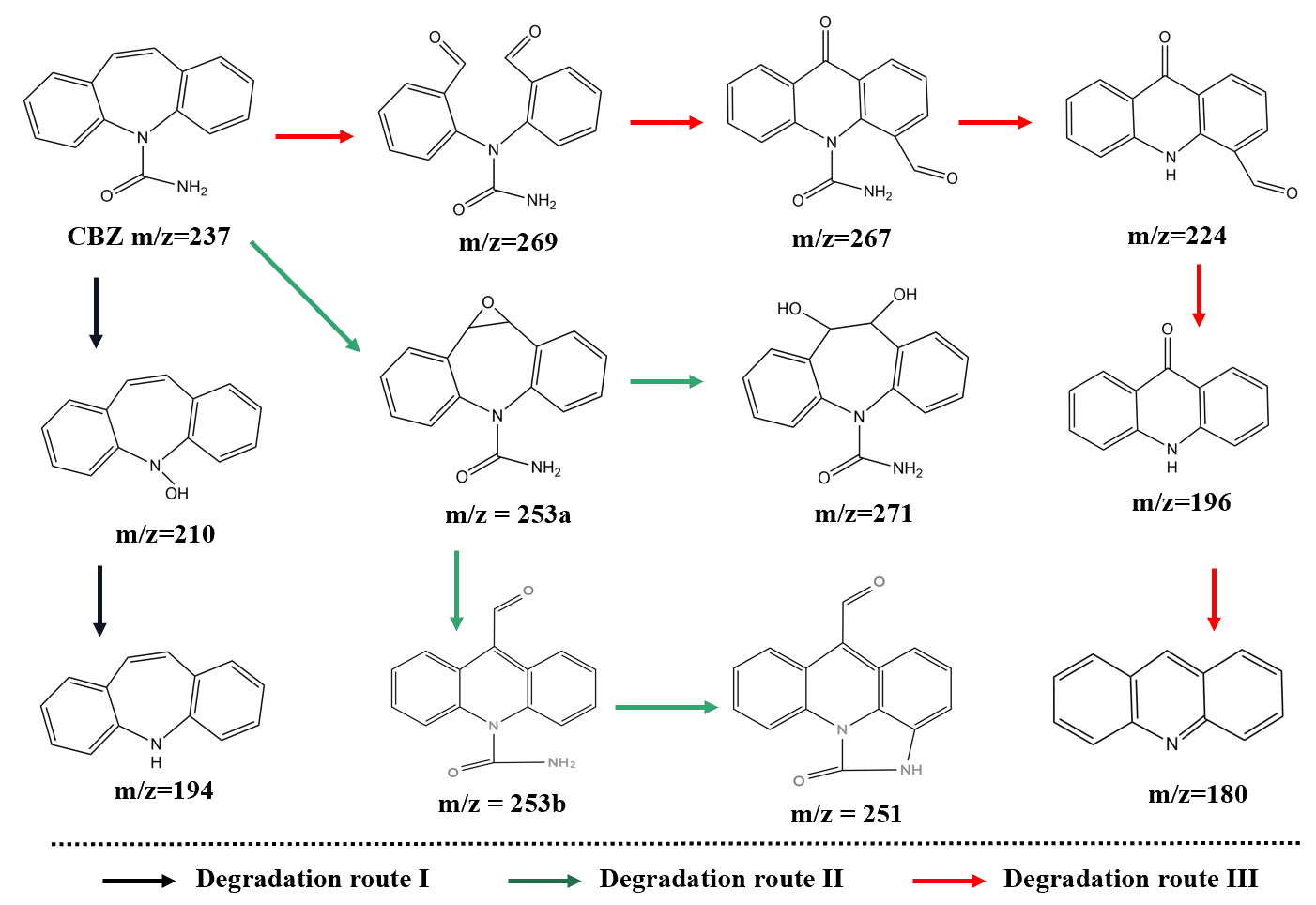
Figure S5. Degradation pathways of CBZ by the PI/PDS/solar light process

Table S1. Detailed experimental conditions of different processes

| Processes | PI dosage | PDS dosage | Light intensity | Solution volume | Solution pH | Temp.  (°C) | Pollutant Conc. |
| --- | --- | --- | --- | --- | --- | --- | --- |
| PI/solar light | 0.5 mM | - | 1008 ± 24  W/m^2^ | 50 mL | 7.0 ± 0.1 | 20.1 ± 0.5 | 1 ppm |
| PDS/solar light | - | 0.25 mM | 1008 ± 24  W/m^2^ | 50 mL | 7.0 ± 0.1 | 20.1 ± 0.5 | 1 ppm |
| PI/PDS solar light | 0.5 mM | 0.25 mM | 1008 ± 24  W/m^2^ | 50 mL | 7.0 ± 0.1 | 20.1 ± 0.5 | 1 ppm |

| Processes | Materials | Pollutant conc. | Working conditions | | | Removal efficiency | Energy consumption | Ref. |
| --- | --- | --- | --- | --- | --- | --- | --- | --- |
|  |  |  | Chemical & conc. | Light source | Time (min) |  |  |  |
| UV/PMS | - | 5 ppm | 1 mM PMS | UV lamp | 90 | 76.2% | 4.67 kWh m^−3^ | Deng et al., 2013 |
| UV/H_2_O_2_ | - | 5 ppm | 1 mM H_2_O_2_ | UV lamp | 90 | 96.22% | 1.98 kWh m^−3^ | Deng et al., 2013 |
| UV/PDS | - | 5 ppm | 1 mM PDS | UV lamp | 90 | 98.91% | 1.49 kWh m^−3^ | Deng et al., 2013 |
| UV/IO_3_^−^ | - | 5 ppm | 0.3 mM IO_3_^−^ | UV lamp | 30 | 92.47% | 9 kWh m^−3^ | Zhang et al., 2022 |
| Photocatalysis/PDS | MoS_2_/rGO/WO_3_ | 2.4 ppm | 0.25 mM PDS | AM 1.5 G solar light | 20 | 88.25% | - | Cui et al., 2021 |
| UV/TiO_2_/PI | TiO_2_ | 5 ppm | 0.05 mM PI | UV lamp | 30 | 87% | 5.57 kWh m^−3^ | Zhang et al., 2022 |
| UV/PDS |  | 4.8 ppm | 0.2 mM PDS | UV lamp | 60 | 100% | - | Zhang et al., 2015 |
| PI/PDS/solar light |  | 1 ppm | 0.5 mM PI, 0.5 mM PDS | AM 1.5 G solar light | 15 | 100% | - | This study |

Table S2. Comparison of different processes in CBZ degradation

**References**

Cui J, Zhang Y, Zheng Y, Pei Y, He X, Xi B (2021) Catalytic degradation of carbamazepine by a novel granular visible-light-driven photocatalyst activating the peroxydisulfate system. Chemical Engineering Journal 421: 127867.

Deng J, Shao Y, Gao N, Xia S, Tan C, Zhou S, Hu X (2013) Degradation of the antiepileptic drug carbamazepine upon different UV-based advanced oxidation processes in water. Chemical Engineering Journal 222: 150-158.

Zhang X, Kamali M, Uleners T, Symus J, Zhang S, Liu Z, Costa MEV, Appels L, Cabooter D, Dewil R (2022) UV/TiO_2_/periodate system for the degradation of organic pollutants–Kinetics, mechanisms and toxicity study. Chemical Engineering Journal 449: 137680.

Zhang X, Kamali M, Yu X, Costa MEV, Appels L, Cabooter D, Dewil R (2022) Kinetics and mechanisms of the carbamazepine degradation in aqueous media using novel iodate-assisted photochemical and photocatalytic systems. Science of The Total Environment 825: 153871.
